# Efficacy and Safety of Modified Double-snare-assisted Endoscopic Mucosal Resection for Small Rectal Neuroendocrine Tumours: A Preliminary Study

**DOI:** 10.1055/a-2938-5538

**Published:** 2026-10-12

**Authors:** Xiwei Ding, Tianlu Huang, Fan Zhou, Supeng Wang, Wei Zhang, Nina Zhang, Liangliang Shi, Dehua Tang, Ying Lv, Lei Wang

**Affiliations:** 1 Department of Gastroenterology 66506 Nanjing University Medical School Affiliated Nanjing Drum Tower HospitalNanjingJiangsuChina

**Keywords:** endoscopy lower GI tract, polyps/adenomas/…, endoscopic resection (polypectomy, ESD, EMRc, …), endoscopic ultrasonography, subepithelial lesions

## Abstract

**Background** Modified double-snare-assisted endoscopic mucosal resection (mDS-EMR) through single-channel endoscopy has been reported as a promising technique for treating rectal neuroendocrine tumours (NETs). We evaluated the efficacy and safety of mDS-EMR for small rectal NETs.

**Methods** This retrospective study included consecutive patients who underwent mDS-EMR between January 2025 and December 2025 at a single centre. Clinical data and therapeutic outcomes were analysed.

**Results** Thirty-five patients with 38 rectal NETs were included. The median lesion size was 5 mm (range: 1–13 mm). The technical success rate was 100%. R0 resection was achieved in 37 out of 38 lesions (97.4%), including the largest lesion. The 95% confidence interval for R0 resection rate is 86.5–99.5%. The en bloc resection rate was 97.4%. The median resection time was 2.5 min per lesion (range: 1–9 minutes). The delayed bleeding rate was 2.6% (one patient). Perforation did not occur during or after the procedure. The median length of hospital stay after the procedure was 2 days (range: 0–5 days). The median cost of the device used for mDS-EMR is $237.4 (range: $186.7–537.2).

**Conclusions** mDS-EMR appeared to be a safe, and effective method for resecting small rectal NETs.

## Introduction

Rectal neuroendocrine tumours (NETs) are malignant tumours that are at risk of metastasis. A Japanese study by Inada et al. revealed that the rate of lymph node metastasis of rectal NETs within 1 cm is approximately 3%. 1 A large sample study from the United States revealed that the lymph node metastasis rate of rectal NETs within 1 cm is 2.5%. 2 Therefore, rectal NETs should be removed even when the lesion is small. Current guidelines recommend local resection of small low-grade rectal NETs (G1/G2 grade) with no signs of metastasis. 3

Endoscopic mucosal resection (EMR) is the most common and simple resection technique for treating colorectal tumours. However, owing to the shallow resection depth, the traditional EMR method has a high rate of positive resection margins for rectal NETs. 4 5 6 Therefore, the traditional EMR method is not recommended for the treatment of rectal NETs. Instead, modified EMR techniques such as cap-assisted EMR (EMR-C) or ligation-assisted EMR (EMR-L) have been shown to result in high R0 resection rates for rectal NETs. 7 8 9 10 11 12 Endoscopic submucosal dissection (ESD) can also obtain high rates of complete resection, with the R0 resection rate reaching 92.7–97.5%, and is currently widely used as a resection technique for rectal NETs. 7 13 However, traditional ESD is relatively time-consuming and costly compared with modified EMR techniques. Additionally, ESD requires more professional skill training.

We previously reported a modified single-channel double-snare-assisted EMR method (mDS-EMR) that can successfully remove small rectal NETs without adverse events. 14 mDS-EMR allows more submucosal tissue to be captured, resulting in improved vertical resectability. In addition, the specific method requires only two small snare devices and conventional single-channel endoscopy. Compared with other modified EMR techniques, mDS-EMR seems more convenient to carry out at each endoscopy centre. In this study, data from our centre were retrospectively analysed to comprehensively evaluate the efficacy and safety of the mDS-EMR for small rectal NETs.

## Patients and Methods

This retrospective study was conducted at a single hospital (Nanjing Drum Tower Hospital, Nanjing, China). Consecutive patients with suspected rectal NETs who underwent mDS-EMR in our hospital between January 2025 and December 2025 were reviewed. The study protocol was reviewed and approved by the Medical Research Ethics Committee of Nanjing Drum Tower Hospital, Nanjing University (2025-1041-01). Informed consent to undergo the procedure was obtained from all included patients.

Patients were eligible for mDS-EMR if they met the following inclusion criteria: (1) an endoscopic diagnosis of a NET ≤10 mm; (2) no obvious tumour invasion into the muscularis propria; and (3) no evidence of metastasis on preoperative computed tomography. Exclusion criterion: lesions whose final pathology after resection was not NET.

All procedures were performed by experienced endoscopists who had performed ≥500 colorectal EMR prior to the study. All procedures were done with single-channel Olympus endoscopes (GIF-H290T; Olympus, Tokyo, Japan), Fujifilm endoscopes (ELUXEO 7000; Fujifilm, Tokyo, Japan), or Aohua endoscopes (UHD-GT300T; Aohua, Shanghai, China). Snares and clips were obtained from Nanjing Micro-Tech (Nanjing, China) and AGS MedTech (Hangzhou, China). The mDS-EMR procedure involved the following steps ( Fig. 1 **,** Fig. 2 ) and has been reported previously 14 : (1) A polypectomy snare (resection snare) was placed outside of the endoscope. (2) After the lesion was approached, a second polypectomy snare (capture snare) was inserted through the biopsy channel of the endoscope to grasp and lift the lesion. (3) The snare preloaded outside of the endoscope was released, passed through the capture snare and positioned below the capture snare to grasp the base of the lesion. (4) Once the lesion was securely grasped, it was resected by a resection snare with the ENDO CUT Q mode of VIO300S or VIO300D (ERBE, Tubingen, Germany) to achieve en bloc resection. (5) Exposed vessels were coagulated using the tip of the snare or hot haemostatic forceps. The area was carefully examined to ensure that no active bleeding occurred. (6) The resection base was closed with clips.

**Fig. 1:**
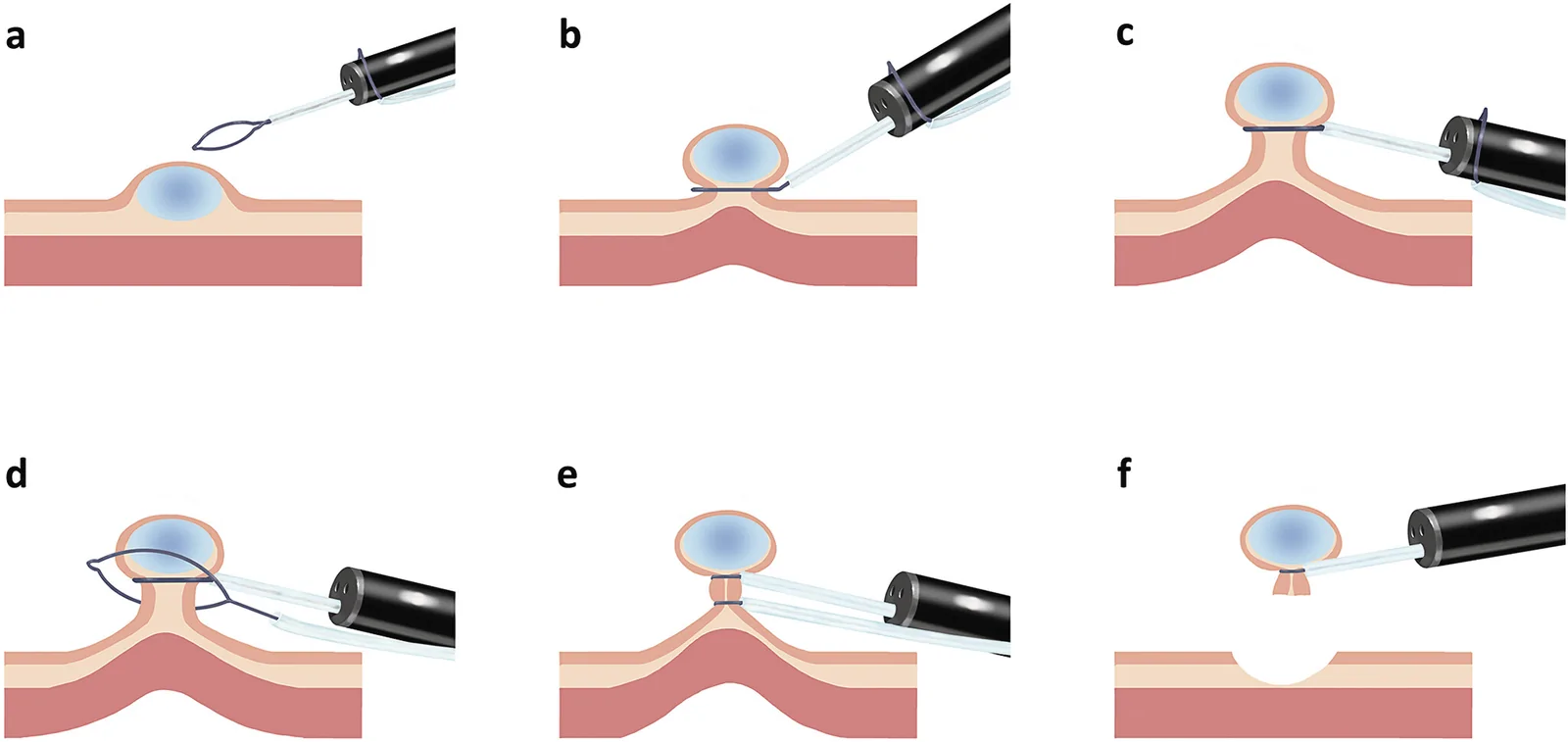
Schematic representation of modified double-snare-assisted endoscopic mucosal resection. ( **a** ) The resection snare was placed outside the endoscope. ( **b** , **c** ) The capture snare was inserted through the endoscope channel to grasp and lift the lesion. ( **d** , **e** ) The resection snare was released and placed beneath the base of the lesion. ( **f** ) The lesion was resected by a resection snare.

**Fig. 2:**
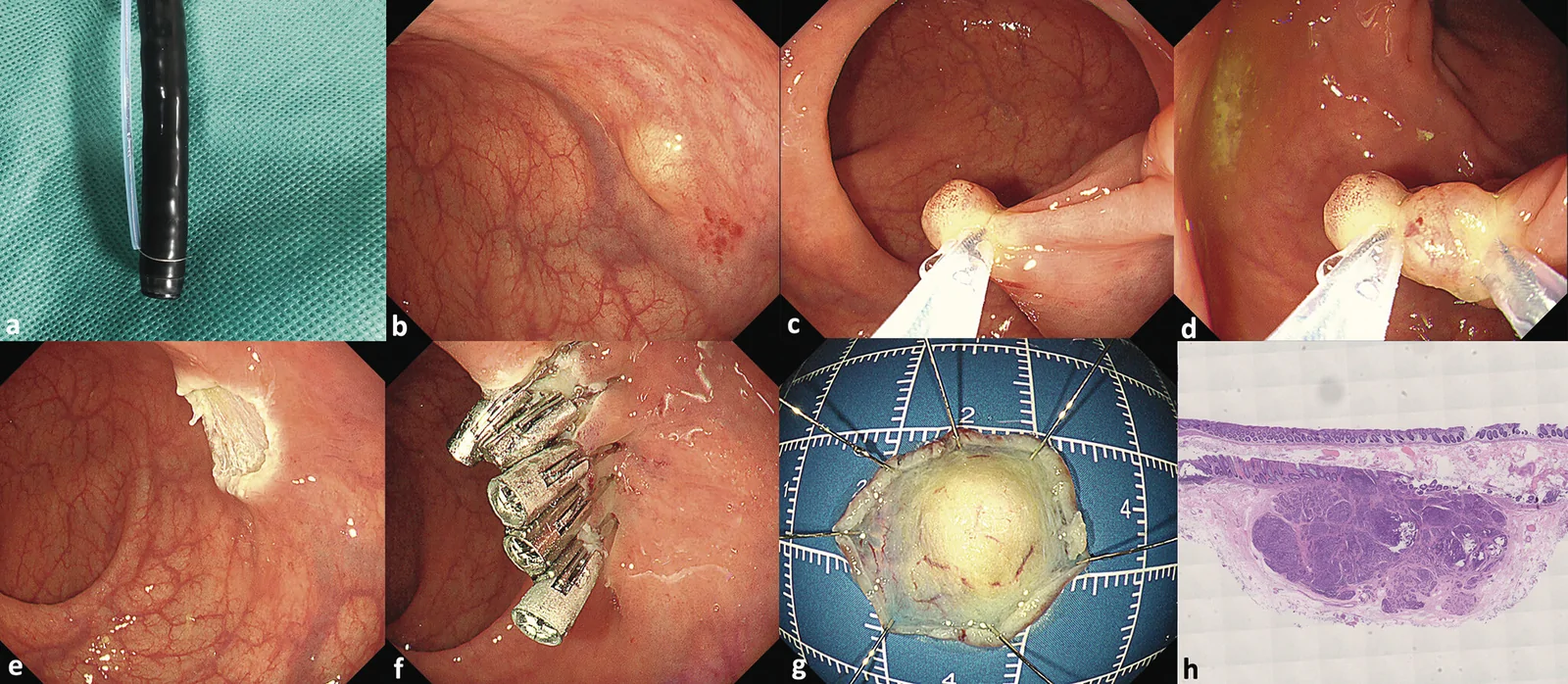
Modified double-snare resection technique for treating a rectal neuroendocrine tumour. ( **a** ) The resection snare was preloaded outside the endoscope. ( **b** ) The tumour in the rectum appeared as a yellowish subepithelial tumour-like lesion whose surface was covered with normal mucosa. ( **c** ) The lesion was lifted by the capture snare. ( **d** ) The resection snare was placed around the base. ( **e** ) The clean base can be observed. ( **f** ) The base was sutured with metal clips. ( **g** ) The specimen was retrieved and sent for pathological evaluation. ( **h** ) Pathology confirmed the NET with negative margins.

Several points should be noted when the mDS-EMR procedure is performed. First, the use of a transparent cap is optional. If the lesion is located in an inconvenient position and difficult to expose, the use of a transparent cap is recommended. In that case, the resection snare is prefixed outside the transparent cap. Second, some flat lesions may not be easily captured by the capture snare. We suggest suctioning the air appropriately to make the lesion more protuberant before it can be captured. Third, the two snare devices cannot be close to each other. Otherwise, the capture snare device may also conduct electricity when the resection snare is powered on, resulting in non-en bloc resection. If we cannot guarantee the distance of the two snares, we suggest releasing the capture snare before resection. Fourth, as there are two snares, it is best to have two assistants control each snare separately. Before powering on for resection, it is important to confirm that the resection snare, not the capture snare, is connected to the high-frequency power source.

The specimen was flattened, fixed in a 10% formalin solution, and sent for pathological examination. The Ki-67 index was estimated to classify the tumour grade into G1, G2, and G3 according to the 2019 World Health Organization classification of digestive tumours.

## Definitions

Technical success was defined as the completion of mDS-EMR without abortion or conversion to other EMR or ESD procedures. En bloc resection was defined as resection of the lesion in a piece with a macroscopic tumour-free margin. R0 resection was defined as complete en bloc resection with microscopically negative margins. Resection time was defined as the duration from the initial fixation of the snare to endoscopy to complete resection of the lesion. Adverse events included postoperative bleeding, perforation during the procedure, and postoperative perforation. Postoperative bleeding was defined as haemorrhage occurring within 30 days after the procedure that required haemostasis. Intraoperative perforation was defined as a defect in the muscular layer observed during the procedure. Delayed perforation was defined as bowel perforation within 30 days after the procedure without intraoperative perforation. EMR-related costs were defined as the total costs of surgical consumables associated with mDS-EMR, excluding any additional endoscopic procedures performed concurrently.

### Statistical Analysis

Statistical analyses were performed using GraphPad Prism 10.0. Continuous variables are expressed as medians and ranges. Categorical variables are expressed as counts and percentages. The 95% confidence interval (CI) of R0 resection rate was calculated using the Wilson score method.

## Results

### Clinicopathological Characteristics

A total of 38 patients with 41 suspected rectal NET lesions underwent mDS-EMR during the study period. Three lesions from three patients with pathological submucosal lymphoid tissue hyperplasia in the resected specimens were excluded. A total of 38 lesions from 35 patients were included in the final analysis. Table 1 shows the characteristics of the patients, lesions, and outcomes. There were 20 male and 15 female patients. The ages ranged from 25 to 81 years, with a median age of 44 years. In terms of the Paris classification, 26 lesions (68.4%) were 0-IIa, and 12 lesions (31.6%) were 0-Is ( Fig. 3 ).

**Table 1:** Characteristics of the patients, lesions, and outcomes treated by mDS-EMR.

Parameters	Results
Age, median (range), years	44 (25–81)
Gender, male/female, *n*	20/15
Lesions per patient, *n*	
One lesion	33
Two lesions	1
Three lesions	1
Macroscopic type, 0-IIa/0-Is, *n*	26/12
Lesion size, median (range), mm	5 (1–13)
Tumour location (distance from anus), median (range), cm	6 (2–13)
Preoperative biopsy, *n* (%)	3 (7.9)
Preoperative EUS, *n* (%)	30 (78.9)
Technical success, *n* (%)	38 (100)
En bloc resection, *n* (%)	37 (97.4)
R0 resection, *n* (%)	37 (97.4)
Positive horizontal margins, *n* (%)	0 (0)
Positive vertical margins, *n* (%)	1 (2.6)
Tumour grading, *n* (%)	
G1	36 (94.7)
G2	2 (5.3)
Lymphovascular invasion, *n* (%)	2 (5.3)
Resection time (min), median (range)	2.5 (1–9)
Depth of submucosal resection, median (range), μm	1865 (990–9400)
Vertical margin distance, median (range), μm	240 (0–940)
Muscularis propria inclusion, *n* (%)	0 (0)
Adverse events, *n* (%) *	
Perforation during procedure, *n* (%)	0 (0)
Delayed perforation, *n* (%)	0 (0)
Postoperative bleeding, *n* (%)	1 (2.6)
Postoperative hospital stays (days), median (range)	2 (0–5)
Hospitalization cost (USD), median (range)	1231.1 (735.7–3953.0)
Costs of devices performing mDS-EMR (USD), median (range)	237.4 (186.7–537.2)

*Including all patients that underwent mDS-EMR.

**Fig. 3:**
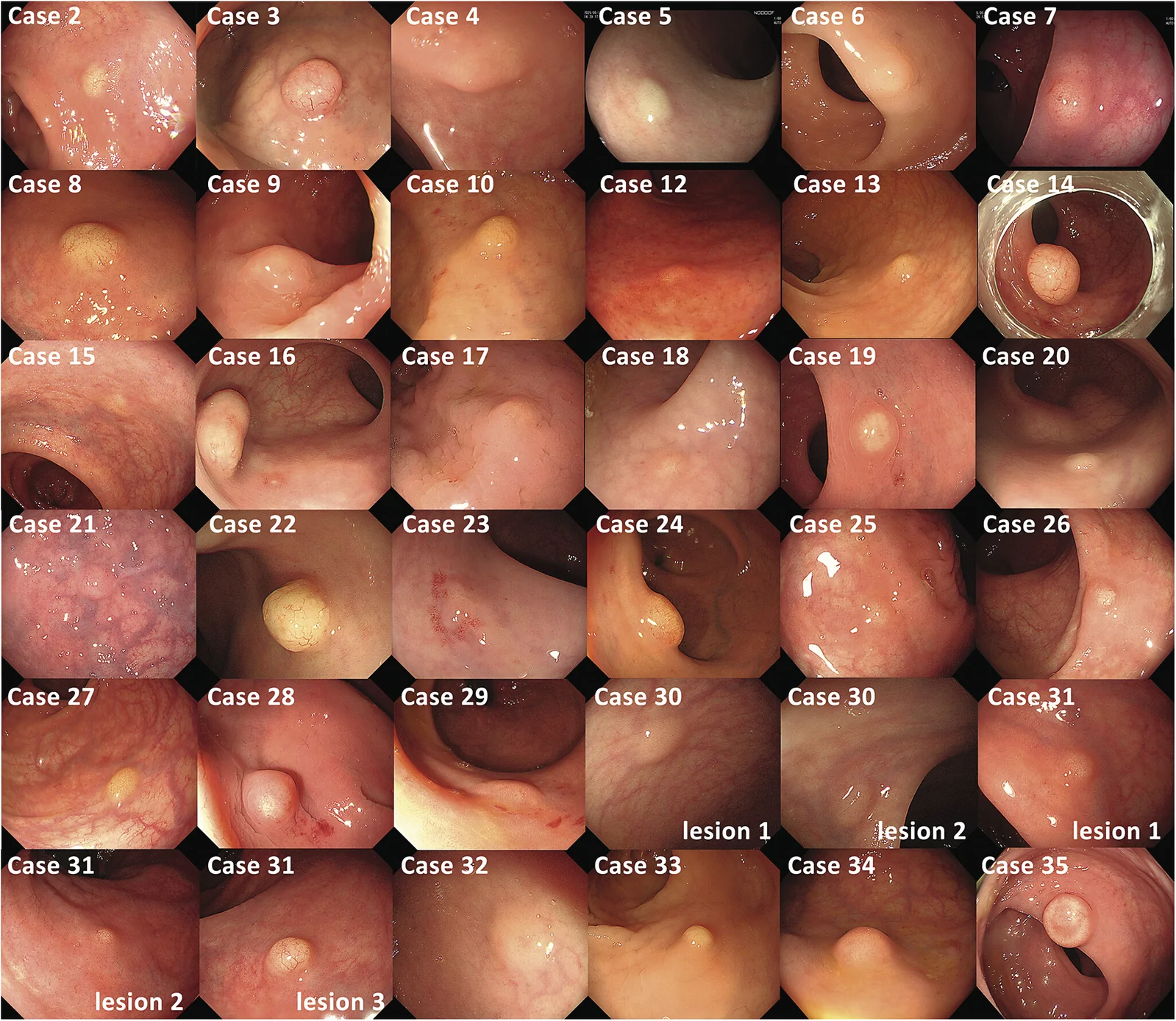
Endoscopic images of 35 patients with 38 rectal neuroendocrine tumours (the image of case 1 was published previously 14 ). An image of case 11 is shown in Fig. 2 . (Therefore, cases 1 and 11 are not shown here.)

### Treatment Outcomes

The median lesion size was 5 mm (range: 1–13 mm) ( Table 1 ). Pathology revealed one lesion larger than 1 cm, although preoperative EUS revealed that the lesion was close to 1 cm in size. Histopathological analysis revealed 36 Grade G1 lesions and 2 Grade G2 lesions. Vascular invasion was detected in two lesions. The technique success rate was 100%. The en bloc resection rate was 97.4%. R0 resection was achieved in 37 of 38 lesions, including the largest lesion. Therefore, the R0 resection rate was 97.4%. The 95% CI for R0 resection rate was 86.5–99.5%. The median resection time was 2.5 min (range: 1–9 min) per lesion. The median depth of submucosal resection was 1865 μm (range: 990–9400 μm). The median distance from tumour front to vertical margin was 240 μm (range: 0–940 μm). The muscle layer resection rate was 0%. The median postoperative hospital stay was 2 days (range: 0–5 days). The hospital stay was 0 for one patient, as she underwent an outpatient operation. The median medical cost during hospitalization was $1231.1 (range: $735.7–3953.0) per patient. The median cost of the device used for mDS-EMR was $237.4 (range: $186.7–537.2) per patient. For adverse event analysis, we included all patients that underwent mDS-EMR, including three patients with non-neoplastic pathology after resection. There were no cases of perforation during the procedure. Postprocedure bleeding related to mDS-EMR occurred in one patient (2.6%). No delayed perforation occurred.

## Discussion

Rojas Sánchez et al. first reported that the DS-EMR technique using a double-channel endoscope is a simple and effective method for removing small rectal NETs. 15 However, double-channel endoscopy is not widely available in endoscopy centres, which may limit its use. The DS-EMR technique through a single-channel endoscope has been shown to be a simple resection method for an oesophageal leiomyoma originating from the muscularis mucosae. 16 We first showed that mDS-EMR through single-channel endoscopy is also a simple, safe, and effective method for resecting a rectal NET. 14 This study reviewed the treatment outcomes of 38 consecutive rectal NET lesions treated by mDS-EMR at our centre, further confirming the effectiveness and safety of mDS-EMR for small rectal NETs. To the best of our knowledge, this is the first case series on the treatment of rectal NETs with mDS-EMR.

EMR-L and EMR-C are commonly used modified EMR techniques for small rectal NETs. The R0 resection rate of mDS-EMR in our study was high and similar to that of other modified EMR techniques. EMR-L requires a ligation device, which increases the treatment cost. EMR-C requires a special cap that is not commonly available. Compared with EMR-L, EMR-C, and other EMR techniques, mDS-EMR only needs two snares and is potentially more cost-effective. The median cost related to the procedure is $237.4. As snare and single-channel endoscopy is available in almost every endoscopy centre, this method may be performed worldwide particularly in settings where double-channel endoscopes or dedicated EMR-L/EMR-C devices are unavailable. Additionally, the resection time is short, and the rate of adverse events is very low. This novel method may also be performed in outpatient settings.

During the study, one case (Case 12) underwent non-en bloc resection. The lesion measured 2 mm in size and was classified as type 0-IIa morphologically. We speculate that the non-en bloc resection occurred because the two snares were positioned too close to each other. After the resection snare was energized, the capture snare also conducted electricity, which resulted in two specimens. Thus, we classified this case as non-en bloc resection. During the mDS-EMR, we should make sure that the two snares not be close to each other. The resection snare should be put away from the capture snare. If the distance of the two snares cannot be guaranteed, we suggest releasing the capture snare before resection.

There are several limitations in the current study. First, this was a single-centre retrospective study. Although we consecutively analysed all patients who underwent mDS-EMR at our hospital during that time and the included patients had different demographic features, lesion locations, and lesion morphologies, the risk of selection bias could not be completely ruled out. Second, as this study focused on short-term outcomes, we could not draw robust conclusions regarding long-term outcomes. Third, this study did not compare mDS-EMR with other modified EMR or ESD methods. Further studies comparing mDS-EMR with other techniques, such as ESD, may be needed. In addition, despite the high observed R0 resection rate, the 95% CI is relatively wide because of the limited sample size. Further accumulation of cases is needed to more reliably establish the resection capability of mDS-EMR. Finally, all mDS-EMR procedures were performed by experienced endoscopists, and the findings cannot therefore be generalized to trainees and less experienced endoscopists.

## Conclusion

mDS-EMR is a potentially safe, and cost-effective method for the resection of small rectal NETs. Large, multicentre, prospective studies are needed to further assess this technique.

## Declaration of GenAI Use

The authors confirm that no generative AI was used in the preparation of this manuscript.
